# Supplementary material for: Structural conservation in a membrane-enveloped filamentous virus infecting a hyperthermophilic acidophile
Source: Nat Commun. 2018 Aug 22;9:3360. doi: 10.1038/s41467-018-05684-6 (PMC6105669; doi:10.1038/s41467-018-05684-6)
Supplement: Supplementary file 1 — Supplementary information [file 41467_2018_5684_MOESM1_ESM.pdf]

Structural Conservation in a Membrane-Enveloped Filamentous Virus Infecting a Hyperthermophilic Acidophile

Ying Liu, Tomasz Osinski, Fengbin Wang et al.

Supplementary Figures

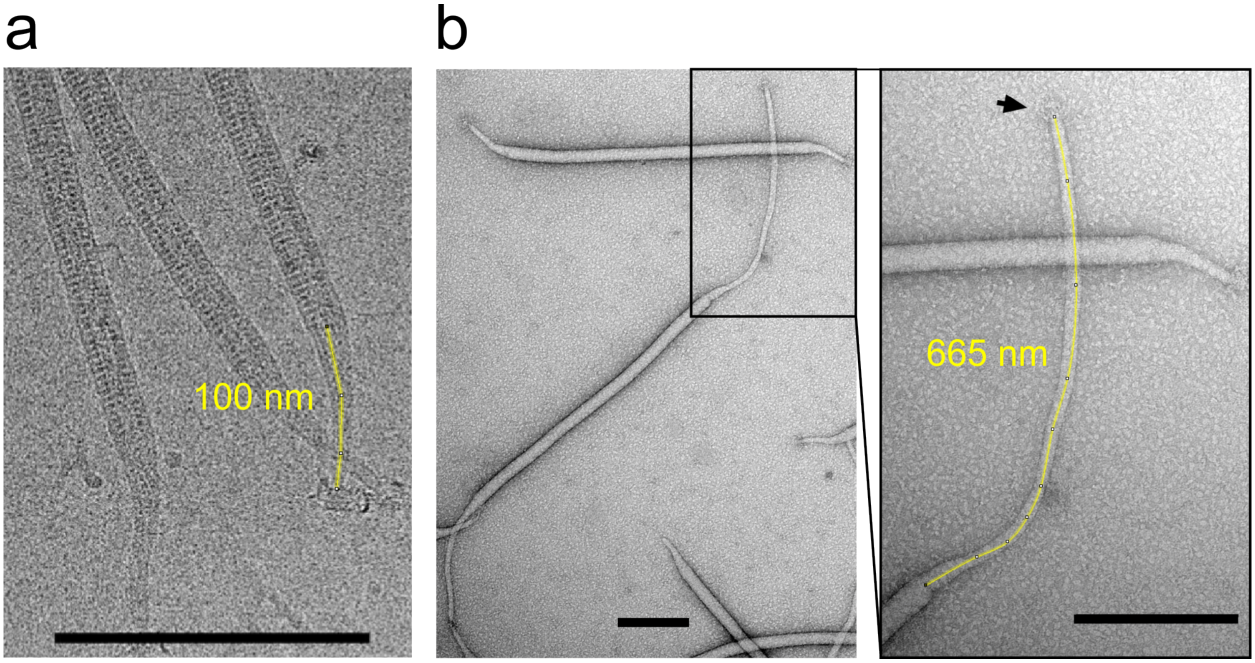

Supplementary Figure 1. Electron micrographs of SFV1, by cryo-EM (a) and negative stain (b). A normal virion neck (a) and a significantly elongated neck (b) are shown. The tail fibers at the terminus of the elongated neck are indicated (arrow). In (b), the sample was negatively stained with 2% (wt/vol) uranyl acetate. Bars, 200 nm.

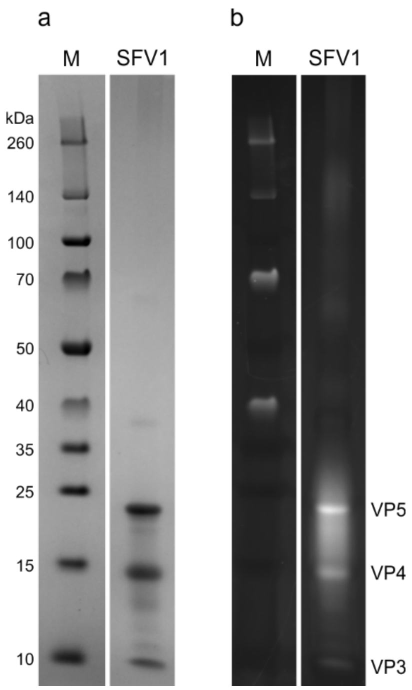

Supplementary Figure 2. Glycosylation of virion proteins was analyzed using ProQ-Emerald Glycoprotein stain. The results indicate that proteins VP3, VP4 and VP5 are glycosylated. SDS-PAGE of SFV1 viral proteins stained with (a) Coomassie Brilliant Blue and (b) with ProQ-Emerald. M, molecular mass standards.

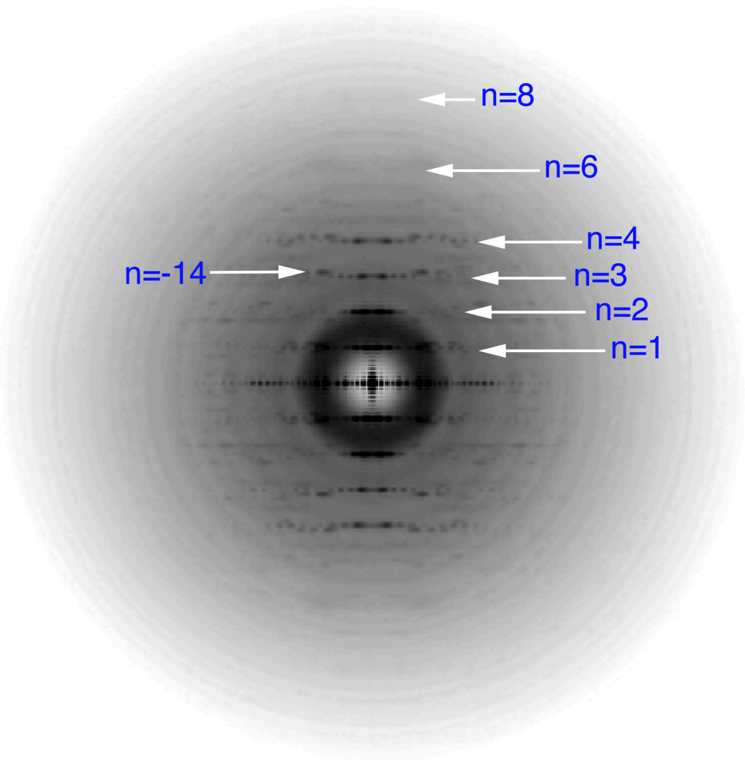

Supplementary Figure 3. A power spectrum generated from 100,379 overlapping segments of SFV1. Multiple orders (from 1 to 8) of the layer line arising from the 47 Å pitch are labeled, as is an n=-14 layer. The n=8 layer line is at a height of 1/(5.9 Å).

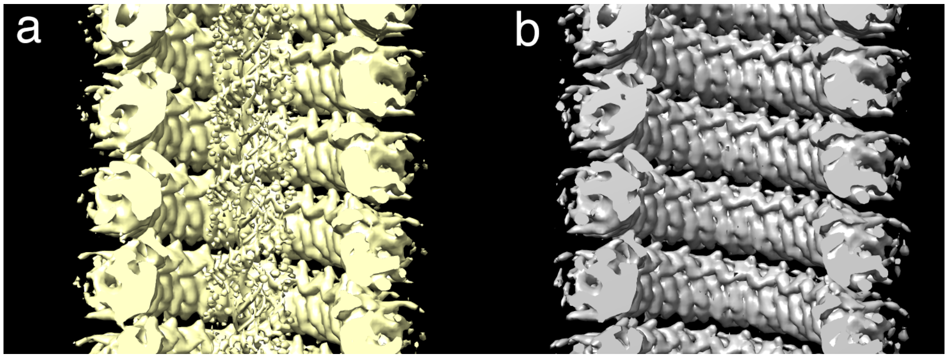

Supplementary Figure 4. Additional density appears in the lumen of the reconstruction at the ends of the virions. Reconstructions were generated from 23,000 segments either at the ends of the virions (a) or from the middle of the virions (b). The volumes have each been filtered to 6 Å resolution, and displayed at similar thresholds.

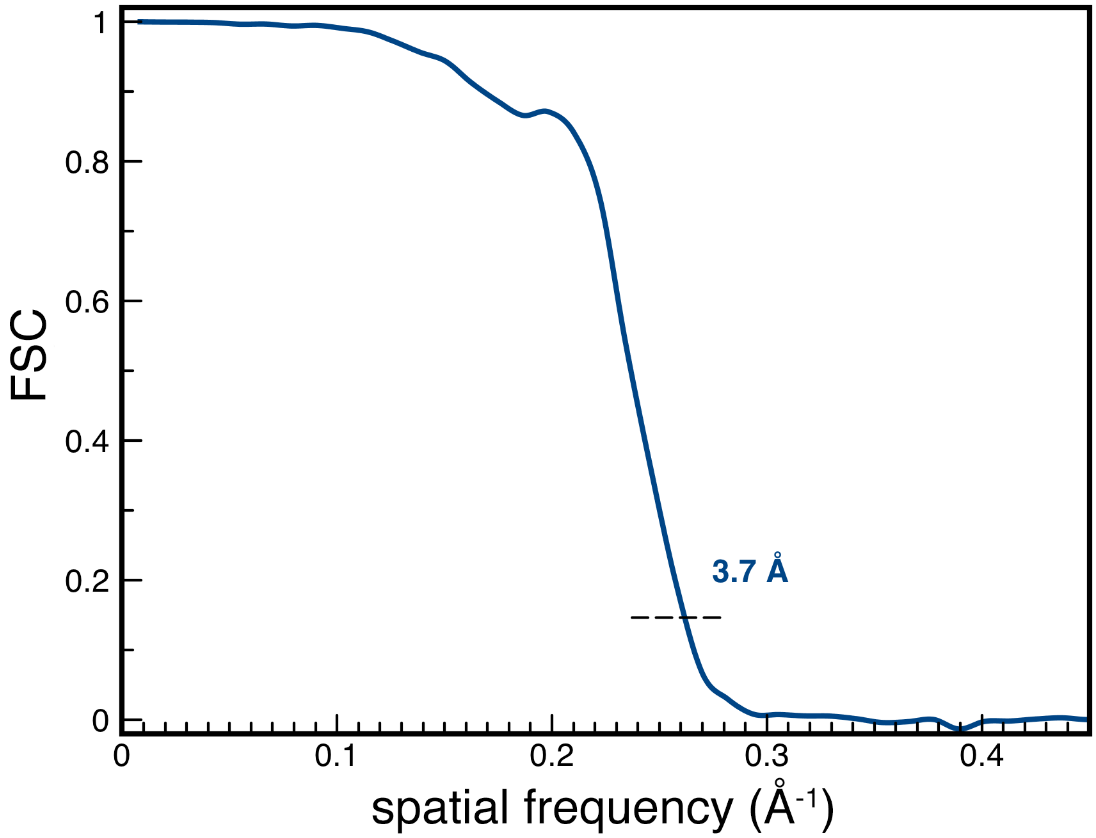

Supplementary Figure 5. The Fourier Shell Correlation between two independent half-maps, each generated from non-overlapping data sets using Relion. Applying the FSC=0.143 criterion, the resolution is 3.7 Å.

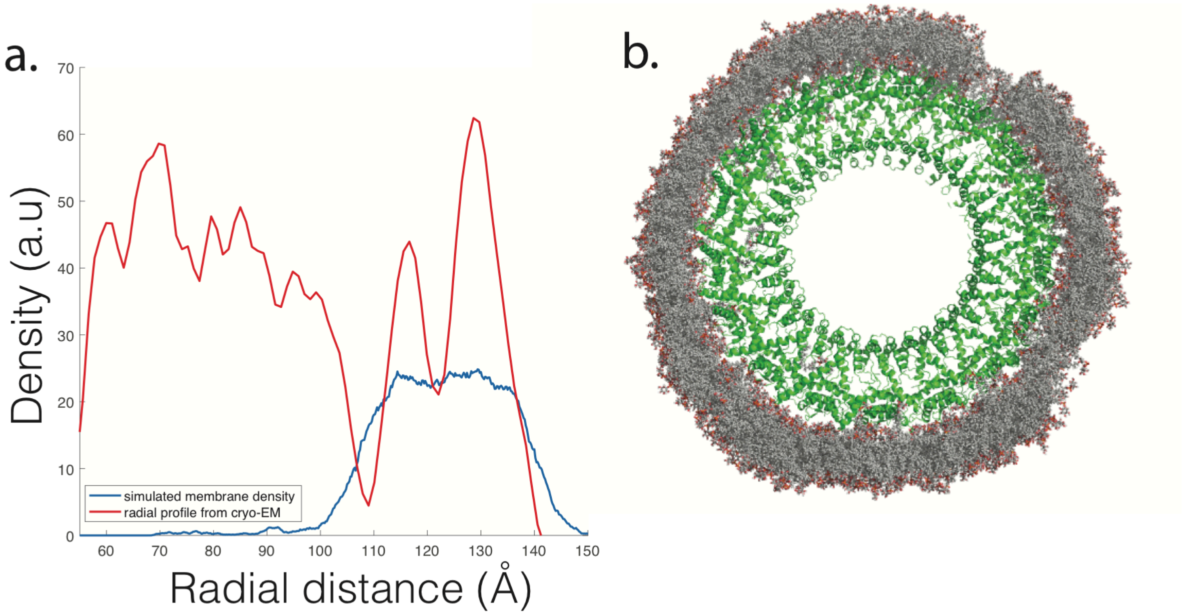

Supplementary Figure 6. (a) The radially-averaged Coulomb potential from cryo-EM versus the radial density profile of phosphatidylinositol archaeol membranes. The membrane approximately reproduces the width of the viral membrane (exceeding it slightly) but does not reproduce the pronounced peaks in the headgroup region, especially that near the outside of the virus. We suggest that these peaks are due to the VP3 viral protein. (b) A simulation of the membrane and viral capsid (snapshot after 81 ns).

Supplementary Table 1: Summary of predicted ORFs in the SFV1 genome

ORF	Coordinates	Length, aa	TMD	Annotation	HHpred hit	Probability	BLAST hit	Identity (%), E-value
ORF1-54	C 153..317	54						
ORF2-617	393..2246	617		helicase	2oca	P=100.0	*Acidianus* filamentous virus 9 (YP_001798539)<br>*Ignicoccus hospitalis* (WP_012123121)	158/606(26%), 3e-35<br>131/459(29%), 3e-36
ORF3-146	2279..2719	146						
ORF4-137a	C 2843..3256	137						
ORF5-115a	C 3249..3596	115						
ORF6-96	3728..4018	96						
ORF7-100a	4005..4307	100						
ORF8-168	C 4317..4823	168						
ORF9-50	C 4833..4985	50					Sulfolobales Virus YNP2 (YP_009216749)	19/42(45%), 0.33
ORF10-175	C 5008..5535	175		glycosyltransferase	2bfw	P=96.8	*Acidianus* filamentous virus 7 (YP_001604264)	26/81(32%), 1.3
ORF11-595	C 5766..7553	595		VP1, ATPase, Bergerat-fold, GHKL superfamily	5ix1	P=98.2	*Edwardsiella* phage KF-1 (YP_006990444)	43/142(30%), 0.058
ORF12-314	C 7541..8485	314		VP2				
ORF13-35	C 8482..8589	35						
ORF14-202	8629..9237	202					*Acidianus manzaensis* (ARM7528)	68/192(35%), 2e-30
ORF15-193a	C 9221..9802	193						
ORF16-292	C 9792..10670	292		glycosyltransferase, GT-B fold	2qzs	P=99.9	*Acidianus* filamentous virus 8 (YP_001604322)	64/279(23%), 0.034
ORF17-118a	C 10648..11004	118						
ORF18-68	11033..11239	68						
ORF19-285	11239..12096	285		glycosyltransferase, GT4 family	2x0d	P=95.42		
ORF20-89	C 12114..12383	89	2	VP3, α-helical protein				
ORF21-308a	12462..13388	308						
ORF22-88a	13372..13638	88						
ORF23-137b	C 13627..14040	137		VP4				
ORF24-199	C 14040..14639	199		VP5				
ORF25-185	14679..15236	185						
ORF26-101	15242..15547	101		RHH	2gpe	P=98.0	*Sulfolobus* monocaudavirus SMV3 (YP_009226286)	21/60(35%), 0.16
ORF27-60	C 15539..15721	60						
ORF28-205	C 15721..16338	205					*Sulfolobus islandicus* filamentous virus 2 (AOS58416)<br>*Acidianus manzaensis* (ARM76918)	62/167(37%), 9e-17<br>60/176(34%), 4e-12
ORF29-92	C 16362..16640	92						
ORF30-124	C 16637..17011	124		VP6				
ORF31-134	16992..17396	134						
ORF32-67a	17393..17596	67	2					
ORF33-78	17621..17857	78		VP7, mainly β-stranded				

ORF34-88b	17854..18120	88		VP8, mainly α-helical				
ORF35-67b	18120..18323	67						
ORF36-115b	18324..18671	115						
ORF37-100b	18635..18937	100	2					
ORF38-98	C 18916..19212	98	3				*Sulfolobus islandicus* filamentous virus 2 (AOS58423)	31/100 (31%), 4.8
ORF39-102	C 19199..19507	102						
ORF40-308b	C 19540..20466	308		glycosyltransferase, Rossmann-fold	2r60	P=99.8	*Sulfolobus islandicus* filamentous virus 2 (AOS58399)<br>*Photobacterium aphoticum* (WP_047876249)	89/307(29%), 2e-30<br>61/215(28%), 8e-06
ORF41-219	C 20499..21158	219	1					
ORF42-364	C 21158..22252	364						
ORF43-230	22289..22981	230						
ORF44-118b	22985..23341	118					*Sulfolobus islandicus* filamentous virus (NP_445712)	36/89(40%), 8e-14
ORF45-136	23341..23751	136						
ORF46-233	23714..24415	233	2					
ORF47-114	24457..24801	114						
ORF48-329	24908..25897	329					*Sulfolobus islandicus* filamentous virus 2 (AOS58403)	97/331(29%), 4e-23
ORF49-295	25890..26777	295	1				*Sulfolobus islandicus* filamentous virus(NP_445718)	39/112(35%), 2e-05
ORF50-583	26770..28521	583		phage-related protein	COG5412	P=99.1	*Acidianus* filamentous virus 2 (YP_001496959)	95/351(27%), 3e-14
ORF51-194	28528..29112	194		glycosyltransferase	cd06437	P=99.5	*Acidianus manzaensis* (ARM75278)	83/193(43%), 1e-35
ORF52-504	C 29076..30590	504		helicase	3i5x	P=100	*Acidianus* filamentous virus 7 (YP_001604228)<br>*Paracoccus versutus* (WP_084197311)	90/333(27%), 6e-06<br>86/362(24%), 6e-07
ORF53-150	30668..31120	150						
ORF54-100c	31126..31428	100						
ORF55-180	31445..31987	180						
ORF56-223	31945..32616	223						
ORF57-112	32626..32964	112		zinc finger	2nb9	P=93.0		
ORF58-193b	32961..33542	193		CRISPR-associated nulease, Cas4 family	4r5q	P=99.6	*Desulfurococcus mucosus* (WP_013561662)<br>*Sulfolobus islandicus* rod-shaped virus 2(NP_666553)	47/163(29%), 5e-04<br>32/119(27%), 1.1
ORF59-104	C 33534..33848	104						
ORF60-138	C 33882..34298	138						
ORF61-122	C 34350..34718	122	1					
ORF62-182	C 34735..35283	182	1					
ORF63-57	35282..35455	57						
ORF64-144	35495..35929	144						
ORF65-331	C 35933..36928	331		GDP-mannose 4,6-dehydratase	1rpn	P=100	*Sulfolobus islandicus* (WP_012713130)	196/321(61%), 2e-140
ORF66-54	36995..37159	54						

C, complementary strand; TMD, transmembrane domain; Blast hits shaded by grey are those E-values higher than 1e-03.

Supplementary Table 2 identification of the genes encoding proteins in SFV1 virions by LC-MS/MS

SFV1 protein (ORF)[a]	Number of identified unique peptides	Coverage (%)[b]
VP1 (ORF11-595)	68	80.3
VP2 (ORF12-314)	36	80.6
VP5 (ORF24-199)	23	100
VP4 (ORF23-137)	26	99.3
VP6 (ORF30-106)	5	23.6
VP7 (ORF33-78)<br>VP8 (ORF34-88)	9<br>4	82.1<br>29.5
VP3 (ORF20-88)	5	21.2

a.The protein bands of the viral proteins are shown in Figure 2b.

b.The percentage of the protein sequence covered by identified peptides.

Supplementary Table 3. Refinement statistics for the SFV1 model

	SFV1 model
Helical symmetry	
Rise (Å)	2.76
Rotation (°)	21.03
CTF selected images	559
Total segments	486,642
Resolution (Å)	3.7
Model to map CC	0.84
Clash score, all atoms	3.68
Protein geometry	
Ramachandran favored (%)	91.3
Ramachandran outliers (%)	0
Rotamer outliers (%)	0.3
Cβ deviations > 0.25 Å	0
RMS deviations	
Bond (Å)	0.01
Angles (°)	1.00
Molprobity score	1.68
Molprobity percentile	99th (3.25 - 3.95 Å)
PDB ID	6D5F
EMDB ID	EMD-7797
